# Supplementary material for: Identification and functional analysis of non-coding regulatory small RNA FenSr3 in Bacillus amyloliquefaciens LPB-18
Source: PeerJ. 2023 May 15;11:e15236. doi: 10.7717/peerj.15236 (PMC10194069; doi:10.7717/peerj.15236)
Supplement: Supplemental Information 4 [file peerj-11-15236-s004.zip › KO/CK-vs-T1_map/map00942.html]

KEGG PATHWAY: Anthocyanin biosynthesis - Reference pathway


|  |  |
| --- | --- |
| **Anthocyanin biosynthesis - Reference pathway** |  |

[
Pathway menu
| Organism menu
| Pathway entry
| Show description
| User data mapping
]

Anthocyanidins (aglycones) and anthocyanins (glycosides) are common plant pigments and belong to a structural subclass of flavonoids characterized by a 2-phenylbenzopyrylium unit. They are derived along the flavonoid modification pathways and further separated into three types, pelargonidin, cyanidin, and delphinidin, due to the different number of hydroxyl groups in the phenyl group.

|  |  |  |
| --- | --- | --- |
| Reference pathway | 184% 150% 122% 100% 82% 67% 55% | 图片下载 |
